# Effect of Genistein on reproductive parameter and serum nitric oxide levels in morphine-treated mice

**Published:** 2016-02

**Authors:** Cyrus Jalili, Sharareh Ahmadi, Shiva Roshankhah, MohammadReza Salahshoor

**Affiliations:** 1 *Fertility and Infertility Research Center, Kermanshah University of Medical Sciences, Kermanshah, Iran. *; 2 *Students Research Committee, Kermanshah University of Medical Sciences, Kermanshah, Iran. *

**Keywords:** *Spermatozoa*, *Catsper protein*, *Antioxidants*, *Ca*^*2+*^* channels*

## Abstract

**Background:**

The predominant phytoestrogen in soy and derived products is the isoflavone Genistein. Genistein has antioxidant properties. Morphine is a main psychoactive chemical in opium that can increase the generation of free radicals and therefore it could adversely affects the spermatogenesis.

**Objective:**

The main goal was to investigate whether the Genistein could protect morphine adverse effects on sperm cells viability, count, motility, and testis histology and testosterone hormone and nitric oxide in blood serum.

**Materials and Methods:**

In this study, various doses of Genistein (0, 1, 2, and 3 mg/kg) and Genistein plus morphine (0, 1, 2, and 3 mg/kg) were administered interaperitoneally to 48 male mice for 30 consequent days. These mice were randomly assigned to 8 groups (n=6) and sperm parameters (sperm cells viability, count, motility and morphology), testis weight and histology, testosterone hormone (ELISA method), FSH and LH hormones (immunoradiometry) and serum nitric oxide (griess assay) were analyzed and compared.

**Results:**

The results indicated that morphine administration significantly decreased testosterone (0.03 ng/mg) LH and FSH level, histological parameters, count, viability (55.3%), morphology and motility of sperm cells (1%), testis weight (0.08 gr) and increase nitric oxide compared to saline group (p=0.00). However, administration of Genistein and Genistein plus morphine significantly boosted motility, morphology, count, viability of sperm cells, seminiferous tubules diameter, germinal thickness, testosterone, LH and FSH while decrease nitric oxide level in all groups compared to morphine group (p<0.025).

**Conclusion:**

It seems that Genistein administration could increase the quality of spermatozoa and prevent morphine- induced adverse effects on sperm parameters.

## Introduction

Phytoestrogens are plant-derived chemicals that have the ability to bind and signal through estrogen receptors. The phytoestrogens, possessing estrogen-like biological activity, have been found to influence human and animal reproduction by changing sexual behavior as well as morphology and function of reproductive organs (1). The predominant phytoestrogen in soy and derived products is the isoflavone Genistein, which accounts for approximately two/thirds of soy isoflavone content. Genistein is a non-steroidal phytoestrogen which binds estrogen receptor1 (ESR1) and estrogen receptor 2 (2). Reports of Genistein’s ability to inhibit, angiogenesis, lipid peroxidation, as well as Genistein’s antioxidant properties, support the possibility that Genistein may have anticancer effects (3). 

Changh *et al* investigate the antioxidant and anti-Inflammatory properties of Genistein and showed that Genistein modified hemodialysis membranes and caused significant reduction of reactive oxygen (4). Genistein has a direct effect on function of mature spermatozoa. Martinez- Soto *et al* showed that effect of Genistein on sperm functionality could be of interest for assisted reproduction treatments (5). Opium substance consumption in young people is increased in comparison with last decade. Opioids produce free radicals and cause apoptosis in some cell (6). Morphine (C_16_H_19_NO_3_) is an opioid analgesic drug, and the main psychoactive chemical in opium. Morphine is addictive and cause physiological dependence (7). Morphine often causes hypogonadism and hormone imbalances in chronic users of both genders and has histological effects on male rat germ cells (8). 

Morphine can adversely affects spermatogenesis and this can occur either by directly inhibiting sperms or testicular function or indirectly by impairing the hypothalamic pituitary testicular axis and cause infertility in men (9). Apoptosis increase in epithelial and immune systems cells by morphine (10). Morphine acts as a pro-oxidan and increase the production of free radicals. El-Hage *et al* showed that production of reactive oxygen species (ROS) increased significantly while HIV-1-coexposed cells expose to morphine (11). In the reproductive system, nitric oxide plays an essential role in blood circulation. Germinal cells apoptosis boost along with nitric oxide expression (12). Infertility observed in 10-15% of the couples and have social side effects. About 40% of infertility problems are associated with men (13). Infertility in males has been associated with sperm dysfunctions such as low sperm count, immaturity, abnormality and lack of motility (14). According to a survey some infertile men are drug addicts (15). However, there is a lack of information about protective effects of Genistein against morphine side effects. 

Therefore, the aim of this study was to evaluate the effects of Genistein on damage induced by morphine in reproductive parameters and serum nitric oxide levels in male mice.

## Materials and methods

**Chemicals**

This experimental study was carried out for 9 months in the Fertility and Infertility Research Center, Kermanshah University of Medical Sciences. Genistein (C_15_H_10_O_5_) powder (Merk- Germany) was dissolved in absolute ethanol (C_2_H_5_OH) and diluted by normal saline (0.9%) to prepare different doses. Also, the morphine (C_16_H_19_NO_3_) (Merk- Germany) was diluted by normal saline (0.9%) for administration (16).

**Animal model**

In this study 48 Balb/c male mice purchased from Tehran Razi Institute weighting from 25±2.2 gr were used. All the animals were housed in plastic cages in a room tempreture at 22±2^o^C, under controlled environmental conditions, 12 hr light/dark cycle and free access to water and food. All experimentation was conducted under approval of Ethics Committee of Kermanshah University of Medical Sciences (Certificate No. 1394.42) (14).

**Experimental design and dosage**

Morphine was administered intera-peritoneally as follows: 10 mg/kg once daily within the first day. On days 2-30, Morphine doses increased 2 mg/kg per day (17). Genistein was administered as follows: On days 1-30, Genistein once daily, interaperitoneally injecting (18). Morphine plus Genistein was administered as follows: On days 1-30, Genistein once daily plus morphine, interaperitoneally injecting (17, 18). The same volume of saline was administered. Mice were randomly divided into 8 groups (n=6). 1) Normal saline group (1 ml DW/daily); 2) Morphine treated group; 3) Genistein 1 mg/kg treated group; 4) Genistein 2 mg/kg treated group 5) Genistein 4 mg/kg treated group; 6) Morphine plus Genistein 1 mg/kg treated group; 7) Morphine plus Genistein 2 mg/kg treated group; 8) Morphine plus Genistein 4 mg/kg treated group. 

**Testis weight and hormone estimations**

The animals were anesthetized 24 hr after the last injection. By cardiac puncture method blood from sacrificed mice were collected into sterile collection vials and preserved in the temperature of 37^o^C for 30 min and was centrifuged to obtain the serum (3000 gr for 15 min). Serum samples were directly frozen at -70^o^C until biochemical analyses. Serum testosterone concentrations were measured by ELISA (Abcam 108666, USA) method. Serum levels of LH and FSH were measured by immunoradiometry assay. The serum levels of LH and FSH were determined, using the RADIM (Rome, Italy) kits. 

For FSH kit variation intra-assay coefficient was 5.3%, the variation interassay coefficient was 5.5% and the assay sensitivity was 0.17 ng/ml. For LH kit the variation intraassay coefficient was 6.8%, the variation interassay coefficient was 7.2% and assay sensitivity was 0.2 ng/ml. The testes along with the epididymis carefully were removed, washed in normal saline solution (0.9%), blotted, and weighed separately and the average weights were used (13).

**Preparation of sperm suspension for analyses of different parameters**

The Cauda epididymis from both sides were taken out, minced and incubated in a pre-warmed petri dish containing 10 ml Hams F10 medium containing 0.5% Bovine Serum Albumin and the solutions were incubated at 37^o^C. The spermatozoas were allowed to disperse into the buffer. After 20 min, epididymis Cauda was removed, and suspension was gently shaken to homogenize (14).

**Sperm count**

For sperm counting, 500 μL of prepared epididymal sperm suspensions were diluted with formaldehyde fixative (10% formalin in PBS). Approximately 10 μL from diluted solution was transferred into a haemocytometer using a Pasteur pipette (Thoma, assistant Sondheim/Rhön, Germany) and let to stand for 7 min. Then the settled sperms were counted and evaluated per 250 small squares of a haemocytometer (13).

**Sperm viability**

Viability was assessed by eosin Y staining (5% in saline). Forty micro liter samples of freshly sperm suspension were placed on a glass slide, mixed with 10 μL eosin and observed under a light microscope (×400 magnification). Live sperms remained unstained following staining, whereas, those that showed any pink or red coloration were classified as dead. At least 200 sperm were counted from each sample in ten fields of vision randomly, and the percentage of live sperms was recorded (13).

**Sperm motility**

To assess the percentage of motile sperm light microscope (Olympus Co., Tokyo, Japan) was used at 400× magnification. The suspension was prepared by repipetting and one drop of sperm suspension was placed on a glass slide and covered with a lamella. Sperm motility was divided to 4 levels according to certain criteria: a) quick progressive motility in direct line, b) slow progressive motility in direct or indirect line, c) no progressive motility and d) no motility were counted in several microscopic fields of vision and the percentages of motile and non- motile sperms were obtained. Motility estimates were obtained from 10 different fields in each sample. The mean of the 10 successive estimations was used as the final motility score (14).

**Sperm morphology**

To examine the sperm cells morphology, smear was prepared from the samples. Eosin/ nigrosin stain was used to estimate sperm morphology. To test, one drop of eosin/ nigrosin was added to the suspension and mixed gently. The slides were then viewed under a light microscope at 400× magnification. A total of 300 spermatozoa were analyzed on each slide (19). 

**Griess assay**

Nitric oxide was measured based on Griess colorimetric assay. Accordingly, N-(1- naphthyl) ethylenediamine dihydrochoride (NEED), sulfonamide solutions and nitrite standards were prepared. To measure nitrite concentration in serum, after de-freezing the serum samples, 100 µl of the sample serum was deproteinized by zinc sulfate and transferred to the wells. 100 µl chloride vanadium, 50 µl sulfonamide, and 50 µl NEED solutions were added afterwards. The cells were incubated in the temperature of 30^o^C in darkness. Samples' optical density (OD) was measured by ELISA reader at the wavelength of 540 nm (20).

**Histological analysis**

Histological analysis of the testes was performed. The testes were fixed in neutral buffered formalin 10% dehydrated, and then embedded in paraffin. Thereafter, five- micron thick sections were prepared and at least five slides from each testis were stained with hematoxylin and eosin (HE) for histological assessment. The specimens were examined under Olympus/3H light microscope. More than 20 sections were prepared from each block. The sections numbered 5, 10, 15, and 20 were selected and photographed separately from three random views. Seminiferous tubules diameter and germinal layer thickness were measured by Motic camera and software (Moticam 2000, Spain). The mean of seminiferous tubules diameter (μm) and germinal layer thickness (μm) were determined for each testis (21).

**Statistical analysis**

The Kolmogorov-Smirnov test was used for assessing normal distribution of variables. All the quantitative data were presented as mean±SD. One-way analysis of variance (ANOVA) followed by LSD post-hoc test were performed to determine the statistical significance between different groups using SPSS software package 16.0. P<0.05 was considered significant.

## Results

**Weight of testis**

**Figure 1:**
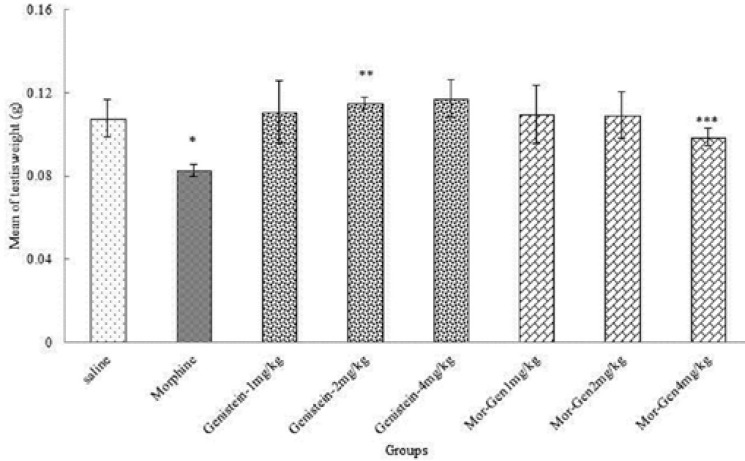
Correlation analysis between treatment groups (morphine, Genistein and Genistein plus morphine) in Balb/c mice and testis weight. * Significant decrease of testis weight in morphine group compared to saline group (p=0.00). ** Significant increase in all groups compared to morphine group (p=0.00). *** Significant increase in all groups compared to morphine group (p=0.028

In the present study, the effective dose of morphine caused a significant decrease in testis weight of mice compared to Saline group (p=0.00). Moreover, testis weight were significantly increase in treated animals with Genistein and Genistein plus morphine in all doses in comparison with morphine group (p=0.028) (Figure 1). 

**Testosterone, LH and FSH hormones**

Morphine caused a significant decrease in the testosterone, LH and FSH hormones compared to saline group (p=0.00). In addition, the testosterone, LH and FSH hormones increased significantly in Genistein (p<0.05) and Genistein plus morphine in all groups administration compared to morphine group (p=0.024) (Table I, Figure II).

**Sperm viability count, morphology and motility**

The sperm viability, count, normal morphology and sperm progressive motility decreased significantly in morphine and Genistein plus morphine (1, 2 and 4 mg/kg) treated groups compared to saline group (p=0.00). Motility, count, normal morphology and sperm viability were significantly increased in Genistein (p<0.05) and Genistein plus morphine in all treated groups in comparison with morphine group administration (p=0.02) (Tables I, II).

**Nitric oxide serum level**

The mean of Nitric oxide in blood serum increased significantly in morphine group administration compared to saline group (p=0.00). Also, the mean of nitric oxide in blood serum decreased significantly in Genistein (p=0.00) and Genistein plus morphine in all groups compared to nicotine group (p=0.00) (Figure 3).

**Histological study**

The morphine administration caused a significant decrease in the seminiferous tubules diameter and germinal layer thickness in comparison with saline group (p=0.00). The Genistein administration (p=0.00) and Genistein plus morphine caused a significant boost in seminiferous tubules' diameter and germinal layer thickness in all groups (p=0.042) (Table I, Figure 4).

**Table I:** Different reproductive parameters between treatment groups in Balb/c mice

** Groups**	**Saline**	**Morphine**	**G**			**G/M**			**p-value**
**Prameters**			**1mg/kg**	**2mg/kg**	**4mg/kg**	**1mg/kg**	**2mg/kg**	**4mg/kg**	
LH (ng/ml)	0.68±0.2	0.41±0.04	1.43±0.5	1.66±0.2	1.15±0.09	0.61±0.2	0.83±0.1	0.18±0.06	22.9±1.3
FSH (ng/ml)	0.96±0.4	0.53±0.1	1.46±0.6	3.01±0.4	1.2±0.1	1.01±0.4	0.9±0.1	0.26±0.06	< 0.01
Seminiferous tubules diameter (µm)	86.3±7.13	77.1±4.32	90.5±6.31	97.4±11.20	94.6±10.25	79.8±2.7	83.6±8.1	82.7±5.2	< 0.01
Germinal layer thickness (µm)	27.26±2.6	17.55±1.19	24.12±0.85	26.17±6.28	25.8±6.3	20.14±3.9	22.64±4.1	22.9±1.3	< 0.01

Independent samples t-test was done as the test of significant. p<0.05 was taken as the level of significant, Data were presented as mean±SD.

G: Genistein

G/M: Genistein plus morphine.

**Table II:** Different sperm parameters between treatment groups in Balb/c mice

** Groups** **Prameters**	**Saline**	**Morphine**	**G**			**G/M**			**p-value**
			**1mg/kg**	**2mg/kg**	**4mg/kg**	**1mg/kg**	**2mg/kg**	**4mg/kg**	
Normal morphology (%)	83.83±2.6	53.16±2.2	82.83±2.6	88 ±2.01	86±1.9	64.16±1.9	68.83±1.5	68.83±1.8	< 0.01
Sperm count (10^6^)	2.78±0.19	0.71±0.1	2.55±0.27	2.05±0.36	1.9±0.14	1.26±0.22	1.2±0.27	1.91±0.15	< 0.01
Fast motility (%)	60.83±1.16	1±0.16	80.83±0.9	100.83±0.4	50.83±1.07	10.5±0.1	10.33±0.4	5±0.2	< 0.01
Sperm viability (%)	75.83±1.1	55.03±3.4	85.75±2.3	89.45±3.09	85.06±1.1	63.95±3.5	67.61±1.2	59.25±3.8	< 0.01

Independent samples t-test was done as the test of significant. p<0.05 was taken as the level of significant, Data were presented as mean±SD.

G: Genistein

G/M: Genistein plus Morphine

**Figure 2:**
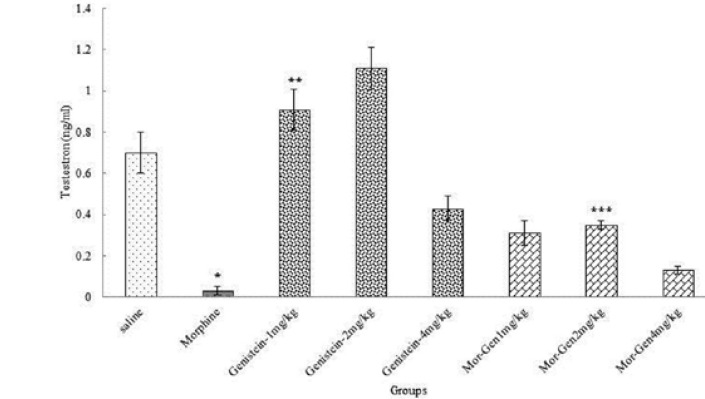
Correlation analysis between treatment groups (morphine, Genistein and Genistein plus morphine) in Balb/c mice and testosterone hormone level. *Significant decrease in morphine group compared to saline group (p=0.00). ** Significant increase in all groups compared to morphine group (p=0.00). *** Significant increase in all groups compared to morphine group (p=0.024

**Figure 3:**
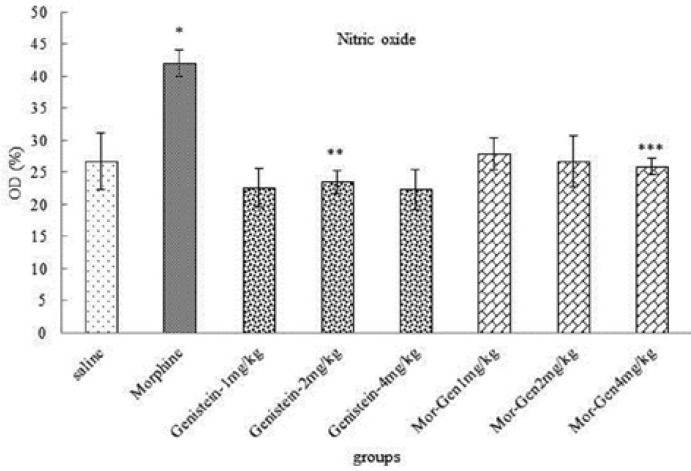
Correlation analysis between treatment groups (morphine, Genistein and Genistein plus morphine) in Balb/c mice and Nitric oxide in blood serum. *Significant increase in morphine group compared to saline group (p=0.00). ** Significant decrease in all groups compared to morphine group (p=0.00). *** Significant decrease in all groups compared to morphine group (p= 0.00

**Figure 4:**
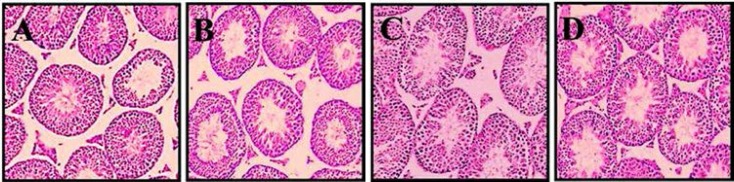
Effect of different concentrations of Genistein on the testis histological. (A) Saline (40×), (B) Genistein (1 mg/kg) (40×), (C) Genistein (2 mg/kg) (40×), (D) Genistein (3 mg/kg) (40×).

## Discussion

Drug addiction is one of problems in current world. It seems that drug use causes changes in sexual function and the hormones associated with them (22). The present study evaluated protective effect of Genistein on reproductive parameters and serum nitric oxide due to morphine administration. Production of free radicals and ROS in sensitive cells of testis seem to have caused reduced testis weight due to morphine administration in current study (23, 24).

The results of Ghowsi *et al* indicate that methadone consumption significantly reduced the weight of sexual organs in mice, which is in line with the findings of present study (17). Increase of seminiferous tubules and number of spermatozoid cells due to anti-oxidant properties of Genistein caused an increase in the weight of testes in this study (25). It seems that morphine induces ROS production and leads to cell cycle arrest and increase of apoptosis, which in turn decreases the daily production and total number of sperms (26). The results of current research showed that Genistein could enhance the sperm count due to proliferation of germinal cells resulting from testosterone increase (25). 

The obtained results confirmed the findings of Selvage *et al* which showed Genistein as a factor to increase testosterone through LH increase (27). However, the findings, were in contrast with results of Bae *et al* which indicated isoflavonid as an inhibitor of testosterone production (28). It seems that ROS reduce the percentage of living sperms by impairing the DNA structure though damaged spermatozoid cells and activated white blood cells in the semen caused by morphine (29). The results of Kubayashi *et al* indicated that regular reduction in number of living sperms is associated with reactive oxygen species increase, which is in agreement with the results of present study (30). 

As a result of excessive ROS, mutations in mitochondrial genome might occur and could disturb the formation of morphologically and functionally mature spermatozoa. It seems that plants such as Genistein, that are rich in antioxidants, protect sperms from damage by free radicals, recover some parameters during a complete spermatic cycle, and improve sperms motility and viability (14). Sperm membranes contain large amounts of unsaturated fatty acids. Morphine-induced oxidative stress cause rapid ATP loss, thereby reducing high motility and sperm viability in the current study (31). 

Genistein, as a potent antioxidant, can protect destructive effects of morphine on sperm viability and motility (32). The findings of current research confirmed the results of Jalili *et al* which showed antioxidants can increase sperm motility (14). Genistein shows estrogen activity and inhibits protein tyrosine kinases. Tyrosine phosphorylation has been reported to have a key role in various aspects of sperm function, one of them being the process of sperm capacitation (33). The parietal cells of seminiferous tubules in groups receiving morphine seem to have been rapidly differentiated and released from tubules, which could reduce the internal diameter of tubules and germinal layer thickness due to morphine administration (13). 

In this study, the germinal epithelium of male mice was decreased by a single dose of morphine. It seems that germ cell apoptosis can be significantly motivated by opoid (34). According to results obtained in present study, Genistein increased and protected the germinal layer thickness and internal diameter of seminiferous tubules compared with the group receiving morphine. This could be also due to high-speed cell differentiation and release of sperm from wall lumens (35). Genistein produced steroid and inhibited protein kinase by bonding to estrogen receptors in lumen of testis, which consequently induced cell proliferation (36). 

The results of Ekinci *et al* study demonstrated that Genistein caused an increase in diameter of seminiferous tubules and germinal layer thickness, which is in agreement with results of present research (37). Morphine could increase the production of nitric oxide by regulating intracellular calcium and activating calcium/calmodulin-dependent NOS. Nitric oxide can reduce sperm motility by reducing ATP production and stimulate apoptosis by impairing the mitochondrial membrane of sperm and releasing cytochrome-C (38). 

Genistein possesses anti-inflammatory effects which decreased nitric oxide in present study via inhibition of prostaglandins and nitric oxide synthesis (39). Some reports suggest that direct effects of opium on pituitary gonadotropine releasing cells. In our study Genistein increased both LH and FSH hormone levels in all treatment groups. Low doses of Genistein seem to increase the secretion of gonadotropin stimulating hormones from hypothalamus, followed by increased LH secretion from pituitary gland and consequently testosterone increase (27). The considerable increase in the FSH level is accompanied by increase in the level of estrogen like chemical, i.e., Genistein which have acted as an agonist of estradiol and it have caused FSH increase, this is similar to the study by Evans *et al* (40). 

Continuously, the significant increase in LH was also in agreement with fact of decrease of Genistein along with the regulation of LH. The results have revealed significant increase in testosterone level in Genistein treated groups, which was in accordance with fact that significant increase in LH has in turn increased the amount of testosterone in testes, therefore increasing spermatogenesis. High level of testosterone was also responsible for increase sperm count, as testosterone is needed for sperm production as well as maturation in the testes (41). 

The findings of the study of Opalka *et al* showed a decline in testosterone level in high concentrations of phytoestrogens, which is in line with the results of current study obtained in groups receiving high concentrations of Genistein (4 mg/kg) (42). The findings of present study indicated that higher dose of Genistein (4 mg/kg) can have a reverse effect on reproductive parameters. Since Genistein contains phytoestrogen, it can affect pituitary-gonadal axis and exert negative effects on testis tissue and secretion of sexual hormones in higher doses (43). Therefore, it seems that phytoestrogens are able to exert both agonistic and antagonistic effects on the studied tissue depending on the dose. Moreover, lower doses of Genistein seem to have more effects on improvement of spermatogenesis parameters and protective damaging effects of morphine. 

## Conclusion

The present study showed that Genistein especially at low doses can significantly improve spermatogenesis in mice. Genistein significantly boosted motility, morphology, count, viability of sperm cells, seminiferous tubules diameter, germinal layer thickness, testosterone, LH and FSH while decrease nitric oxide level in all groups. The results also suggest the protective potential of Genistein especially at low doses studied against toxic effects of morphine-treated male mice. However, further studies are required for a better understanding of the interaction between Genistein and morphine mechanism leading to changes of spermatogenesis.
